# The course of recovery of locomotor function over a 10‐week observation period in a rat model of femoral nerve resection and autograft repair

**DOI:** 10.1002/brb3.1580

**Published:** 2020-02-25

**Authors:** Johannes Christoph Heinzel, David Hercher, Heinz Redl

**Affiliations:** ^1^ Ludwig Boltzmann Institute for Experimental and Clinical Traumatology in the AUVA Trauma Research Center Vienna Austria; ^2^ Austrian Cluster for Tissue Regeneration Vienna Austria

**Keywords:** CatWalk XT, femoral nerve, functional recovery, gait analysis, rats, sciatic nerve

## Abstract

**Background:**

A great extent of knowledge on peripheral nerve regeneration has been gathered using the rat sciatic nerve model. The femoral nerve model of the rat offers an interesting alternative, as it lacks disadvantageous features such as automutilation. For the analysis of locomotor behavior in rats after sciatic nerve injury, the CatWalk^™^ XT Gait Analysis System is often used. However, lesions of the femoral nerve in the rat have yet remained unstudied with this method.

**Material and Methods:**

Ten male Sprague Dawley rats were evaluated with the CatWalk XT to study their gait after a 6‐mm resection of the right femoral nerve and reconstruction with an autologous nerve graft. Animals were observed for 10 weeks after surgery.

**Results:**

Print Area, Print Length, Swing Speed, and Duty Cycle decreased to a minimum of 40% of baseline 2 weeks after surgery. Swing Time was elevated more than twofold at this time point. However, all these parameters recovered back to >90% of baseline values at 10 weeks after surgery. This degree of functional recovery has not been reported after sciatic nerve resection and autograft repair. Base of support varied minimally postoperatively in contrast to a strong decrement after sciatic nerve resection and repair.

**Conclusion:**

We hereby provide a comprehensive in‐depth analysis of how to study functional recovery after injury of the femoral nerve in the rat via the CatWalk XT. We place special emphasis on highlighting the differences between the femoral nerve and sciatic nerve injury model in this context.

## Graphical abstract

Peripheral nerve repair is often studied in the sciatic nerve model of the rat. However, the degree of functional recovery of locomotor function is limited after segmental damage of the sciatic nerve. The femoral nerve model offers an interesting alternative, as functional recovery ad integrum is observable ten weeks after surgery.

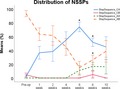

## 1 INTRODUCTION

Rodents, especially rats, are commonly used to study peripheral nerve injuries, given the fact that this animal model is both cost‐effective and a lot of comparative data exist, including data of functional recovery (Deumens, Marinangeli, Bozkurt, & Brook, 2014; Tos et al., 2009). Besides all histological, electrophysiological, and other in vivo and in vitro assessment techniques, assessment of functional recovery is of crucial importance to determine the impact of a certain surgical treatment or medication on peripheral nerve regeneration (Medinaceli, Freed, & Wyatt, 1982). The CatWalk^™^ XT (previously: CatWalk^™^) is a fully automated gait analysis system, which was developed and validated in 85 female rats with spinal cord contusion injury and placed strong emphasis on hind limb–forelimb coordination as essential marker of functional recovery (Basso, Beattie, & Bresnahan, 1995; Hamers, Koopmans, & Joosten, 2006; Hamers, Lankhorst, Laar, Veldhuis, & Gispen, 2001). It was soon discovered that the device was able to record far more data and allows recording of a vast quantity of parameters related to locomotion. These can be further subdivided into three main categories (Table 1): General parameters of gait: Print Area, Print Length, Swing Speed, Base of Support (BoS; the distance between the two hind‐ or front paws), Stride Length (the distance between two consecutive placements of a paw) etc.; pain‐related parameters of gait: Swing Time, Stand Time, and Duty Cycle (Stand Time divided by Stand Time plus Swing Time); coordination‐related parameters of gait: Normal Step Sequence Patterns (NSSP; Cheng et al., 1997; Deumens, Jaken, Marcus, & Joosten, 2007), which categorize the specific sequences of paw placements during a particular step cycle, Phase Dispersions (Kloos, Fisher, Detloff, Hassenzahl, & Basso, 2005), which measure the temporal differences between the step cycles of two specific paws and the Regularity Index (RI), which quantifies the degree of interlimb coordination by dividing the amount of flawless NSSP times 4 by the overall number of paw placement during one step cycle (Deumens et al., 2007; Hamers et al., 2001).

**Table 1:** Analyzed parameters of gait

Parameter of gait	Explanation
General	
Print Area	
Print Length	
Swing Speed	
Base of Support (BoS)	Distance between the two hind or front paws
Stride Length	Distance between two consecutive placements of a paw
Pain related	
Swing Time	
Stand Time	
Duty Cycle	Stand Time divided by Stand Time plus Swing Time
Coordination related	
Normal Step Sequence Patterns (NSSP)	Specific sequences of paw placements during a step cycle
Phase Dispersions	Temporal differences between the step cycles of two specific paws
Regularity Index (RI)	Quantification of interlimb coordination by dividing the amount of flawless NSSP times 4 by the overall number of paw placement during one step cycle

Note

The parameters are sorted into groups of related parameters.

The rat sciatic nerve model is the most frequently used rodent model to investigate peripheral nerve regeneration (Irintchev, 2011) after axonotmesis (Chen et al., 1992; Liskiewicz et al., 2016; Neerven et al., 2012) or neurotmesis (Bozkurt et al., 2016; Madison, Zomorodi, & Robinson, 2000; Varejao et al., 2003). Methods to assess functional recovery in this particular model via gait analysis are quite numerous (Bozkurt, Tholl, et al., 2008; Lin, Pan, Hom, Sabbahi, & Shenaq, 1996; Monte‐Raso, Barbieri, Mazzer, Fonseca, & Barbieri, 2010; Varejao, Melo‐Pinto, Meek, Filipe, & Bulas‐Cruz, 2004), but evaluation of locomotor ability via CatWalk is one of the most frequently used methods (Bozkurt et al., 2011; Deumens et al., 2007; Vrinten & Hamers, 2003). However, the sciatic nerve defect model has major drawbacks, such as its mixed fiber types. Lesions of the sciatic nerve affect multiple muscles which are working antagonistically (Stromberg & Hebel, 1976). Therefore, misdirection of axons will disturb the very subtle balance of agonists and antagonists, seriously limiting the potential of functional recovery (Haastert‐Talini, 2017; Ruiter, Spinner, Verhaagen, & Malessy, 2014). In consequence, a maximum of 50% of normal function assessed by walking track analysis was reported as superlative potential for regeneration in a model of sciatic nerve transection and interfascicular nerve repair (Nichols et al., 2005). Irintchev pointed out that repair of the sciatic nerve commonly causes neuropathic pain in rodents (Irintchev, 2011). This significantly impairs assessment of gait in these animals (Eaton, 2003; Wall et al., 1979). Another major complication based on this phenomenon is, depending on the rat strain, self‐mutilation after sciatic nerve injury. As a result, these animals must be excluded from further follow‐up functional testing, as results will be restricted in validity (Irintchev, 2011; Kingery & Vallin, 1989; Wall et al., 1979; Weber, Proctor, Warner, & Verheyden, 1993).

An alternative model for studying peripheral nerve repair and regeneration in rodents is the femoral nerve model as proposed by Brushart and colleagues in the 1980s (Brushart & Seiler, 1987). In rats, the femoral nerve is mainly formed by parts of the lumbar nerves II, III, and IV and occasionally by the fifth lumbar nerve. After supplying the muscular innervation to the iliacus muscle and pectineus muscle and proximal to the inguinal ligament, the femoral nerve divides into one sensory ramification, the saphenous nerve and into the muscular branch to the quadriceps muscle of the thigh (Brushart, 1988; Stromberg & Hebel, 1976). Considering the beforementioned anatomy and topography of the femoral nerve in rats, when the site of nerve injury and repair is directly proximal to this site of arborization, there is a 50:50 chance for each regenerating muscular axon to correctly reach the quadriceps muscle or otherwise the skin, which is innervated by the saphenous nerve (Irintchev, 2011). Additionally, the femoral nerve model offers a relatively “simple” type of lesion when compared to the sciatic nerve, as transection of its muscular branch will only affect the quadriceps muscle of the thigh, which is responsible for extension of the knee joint (Irintchev, 2011; Kruspe, Thieme, Guntinas‐Lichius, & Irintchev, 2014; Madison, Robinson, & Chadaram, 2007; Stromberg & Hebel, 1976). In terms of key benefits, the femoral nerve model seems to offer another advantage, which is the missing of feared complications such as autotomy, contractures of the limb or skin ulcerations, but it has yet to be proven that these do never occur or just far less frequent than in the sciatic nerve model (Irintchev, 2011). In the last years, studies of peripheral nerve repair and regeneration in the rat femoral nerve model increased considerably (Adel et al., 2017; Hong, Hong, Gu, Lin, & Yin, 2017; Irintchev, Guntinas‐Lichius, & Irintchev, 2018; Meng et al., 2018; Xia et al., 2015). In contrast to the sciatic nerve model, no satisfactory method had been published to perform functional analysis in the femoral nerve model until 2005 (Irintchev, Simova, Eberhardt, Morellini, & Schachner, 2005). Most studies focused on anatomical and morphological investigations as well as retrograde labeling of motoneurons to quantify nerve regeneration (Irintchev et al., 2005). In a study published recently, our group was the first to use the CatWalk XT device to investigate changes of locomotor function in a rat model of femoral nerve injuries (Hercher et al., 2019). However, in the present study we report in detail the specific changes in gait behavior including step sequence patterns. Furthermore, we show correlation data on an array of parameters to electrophysiological data. Only one other study could be identified which investigated changes of gait after volumetric muscle loss of the quadriceps muscle of the thigh by means of CatWalk (Li, Willett, Uhrig, Guldberg, & Warren, 2014). Print Area and Duty Cycle were significantly reduced in comparison to baseline values at 2 and 4 weeks after injury, while showing strong correlation with the injured leg muscle contractility. Of note, both parameters reached their minimum 4 weeks after injury and data collection ended at this postoperative time point (Li et al., 2014). Therefore, we present the novelty of comprehensive and detailed locomotor assessment in rats with femoral nerve resection and autograft repair with help of the CatWalk XT device. This also includes analysis of gait patterns (NSSP) before and after segmental femoral nerve injury, which has not been published before. Additionally, we evaluated the correlation of electromyographic results with locomotor function.

## 2 MATERIAL AND METHODS

### 2.1 Animals and surgery

The specific surgical protocol has been published recently by our group (Hercher et al., 2019). In brief, ten male Sprague Dawley rats (Animal Research Laboratories), weighing 300–350 g, underwent surgery of the right femoral nerve under an operation microscope (Leica M651; Leica Microsystems). A 6‐mm segment of both, the muscular branch to the quadriceps muscle of the thigh and the sensory branch were removed microsurgically, and both gaps were then bridged with the original nerve segment as a homotopic nerve autograft with two sutures per coaptation site (Ethilon^®^ 11‐0; Ethicon‐Johnson & Johnson). The postoperative observation period lasted 10 weeks (*n* = 10).

### 2.2 Ethical approval

The experimental protocol was approved in advance by the Animal Protocol Review Board of the City Government of Vienna. All procedures were carried out in full accord with the Helsinki Declaration on Animal Rights and the Guide for the Care and Use of Laboratory Animals of the National Institutes of Health.

### 2.3 Functional analysis

Functional analysis was performed with the CatWalk XT (Version 10.6) automated gait analysis system (Noldus Information Technology). The animals were accustomed to the CatWalk XT Gait System at least once before performing baseline evaluation and surgery and were examined postoperatively with intervals of 5–7 days. Rats were trained to cross the walking track with a speed between 50 and 100 cm/s, in accordance with relevant publications (Koopmans et al., 2007). We collected three runs per trial at baseline and from then on, every week until 10 weeks after surgery. Data were collected according to the established standards as recommended in the literature (Deumens et al., 2014; Koopmans et al., 2007). The following parameters were assessed (Table 1): BoS (mm), Stride Length (mm), Print Length (mm), Print Area (mm^2^), Swing Speed (s), Stand Time (s), Duty Cycle (%), Swing Time (s), Regularity Index (%), distribution of Normal Step Sequence Patterns (NSSP) (%), Phase Dispersions (%). All parameters with exception of BoS and the last three were assessed for both hind paws, and for each one, a ratio was calculated by dividing the right side's value (resection and homologous autograft repair) by the left side's (control). This ratio was then compared with the ratio at baseline for each postsurgical time point and the result given in per cent. All data in this work were calculated and graphed as mean ± standard error of the mean (*SEM*).

### 2.4 Electrophysiological analysis

In order to evaluate successful reinnervation of the quadriceps muscle, compound muscle action potential (CMAP) and peak amplitude (hereafter referred to as amplitude) of the voltage signal were measured at the end of the observation time at 10 weeks. The femoral nerve was explored, and for stimulation of the motor branch, a bipolar stimulation electrode was placed proximal to the bifurcation using a micromanipulator. Two‐needle electrodes were placed into the quadriceps muscle approximately 10 mm apart for recording, whereas the grounding electrode was placed in the surrounding tissue. A Neuromax EMG device (Natus) was used for stimulation and recording. The contralateral healthy femoral nerve and quadriceps muscle served as an internal control. Core temperature of the animal was measured rectally and used for normalization.

### 2.5 Statistical analysis

All statistical analyses were performed using IBM SPSS version 24 (International Business Machines Corporation). For the CatWalk data, means of all parameters of the defined group were tested for statistically significant differences at each measured postoperative time point using repeated measures ANOVA (RMANOVA). We used Bonferroni correction to adjust the level of significance, and *p*‐values of .05 or lower were considered statistically significant.

For the analysis of correlation between the CatWalk data and electrophysiological data, Print Area, Print Length, Swing Time, BoS, Duty Cycle, and Swing Speed were correlated to both peak amplitude and CMAP at WPO10 using linear regression analysis with IBM SPSS version 24. We decided to perform this regression analysis with the general and pain‐related parameters only. As the coordination‐related parameters of Regularity Index, NSSP and Phase Dispersions are related to all four paws, correlating them with electrophysiological measurements of one paw alone would in our opinion be of reduced validity. *p*‐values of .05 or lower were considered statistically significant.

## 3 RESULTS

### 3.1 **Data quality (**Data S1 **) and Animal welfare**

For statistical analysis of the CatWalk data, three animals had to be excluded due to not evaluable data at least three time points, including WPO10. Two additional animals had to be excluded due to partially not evaluable data at WPO8. Given this, we tested the observed power for each parameter of gait with G*Power (version 3.1.9.4). This program provides the feature of post hoc power analysis for ANOVA with repeated measures (Faul, Erdfelder, Buchner, & Lang, 2009; Faul, Erdfelder, Lang, & Buchner, 2007). All required data for this calculation could be acquired from the original SPSS data output file. Post hoc power‐testing revealed that observed power was below 0.8 for the parameters Stride Length and Regularity Index.

For linear regression analysis, the three animals with not evaluable data at WPO10 had to be excluded, but we included the two animals with missing data at WPO8, since we correlated CatWalk data to electrophysiological data at WPO10 only.

There were no cases of limb automutilation or contractures during the observation period.

### 3.2 CatWalk XT

#### 3.2.1 General gait parameters (Figure 1a–d)

**Figure 1:**
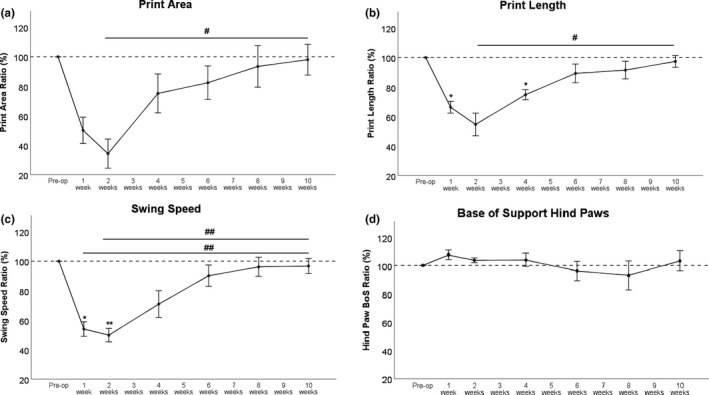
Print Area (a), Print Length (b), and Swing Speed (c) were all clearly affected postoperatively. Minimal values were reached WPO2 but started to increase at WPO4. All parameters returned to their preoperative values at WPO10. BoS (d) was slightly increased initially. *p < .05 as compared to preoperative values. **p < .01 as compared to preoperative values, ^#^ *p* < .05; ^##^ *p* < .01. BoS, Base of Support

Print Area (Figure 1a), Print Length (Figure 1b), and Swing Speed (Figure 1c) decreased at WPO1. However, this was statistically significant only for Print Length and Swing Speed. Lowest values were reached WPO2. While this was highly statistically significant compared to preoperative values in terms of Swing Speed, Print Area at WPO2 showed also a strong trend with *p* = .055. All parameters were increased again at WPO4 compared with lowest values reached WPO1, showed an even further increase at WPO6 and reached >95% of baseline at WPO10. The increase from WPO2 to WPO10 was statistically significant for Print Area and Print Length (*p* < .05) and highly statistically significant for Swing Speed (*p* < .01). In terms of Swing Speed, there was also a highly statistically significant difference between WPO1 and WPO10 (*p* < .01).

Base of Support (Figure 1d) of the hind limbs and Stride Length (data not shown) presented no significant changes during the 10‐week observation period.

#### 3.2.2 Pain‐related parameters (Figure 2a,b)

**Figure 2:**
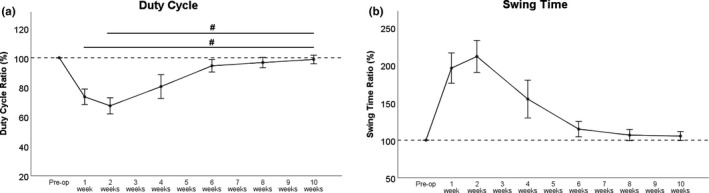
Both, Duty Cycle (a) and Swing Time (b) decreased strongly after femoral nerve resection and autograft repair, with lowest values at WPO2. Both parameters showed an increment at WPO4 and returned close to baseline values at WPO6. Ten weeks after surgery, preoperative values were reached again. ^#^ *p* < .05

Duty Cycle (Figure 2a) decreased at WPO1 and even further the following week. Recovery back to 90% of baseline values was reached WPO6. At WPO10, Duty Cycle was significantly increased (*p* < .05) compared to both WPO1 and lowest values at WPO2. Swing Time (Figure 2b) increased more than twofold of baseline at WPO1. It reached a maximum at WPO2 and deceased toward baseline again at WPO4. Swing Time returned close to baseline values at WPO6. Of note, no statistically significant changes to preoperative values were reached for either parameter.

### 3.3 Coordination‐related parameters

#### 3.3.1 NSSP (Figures 3 and 4)

The course of the six different NSSP over the 10‐week observation period is shown in Figure 4. The Aa pattern was not used before surgery while the Ab pattern was used in 80%. Usage of the Ca pattern was <10%, and usage of the Cb pattern was 5% before surgery. Rats ceased to use Cb immediately after surgery. Ca became the most dominant pattern at WPO3 and reached maximum values at WPO5, compensating the use of the Ab Pattern which was used in <50% of cases from WPO4 onward and reached a minimum at WPO6. Aa started to increase at WPO4 and reached a maximum of 16% at WPO10. At WPO8, usage of Ab increased again, reaching around 50% at WPO10. Concomitantly, the increased percentage of Ca patterns during the walking cycle decreased again after WPO6. There was no use of the Rotary pattern a or b at any time point.

**Figure 3:**
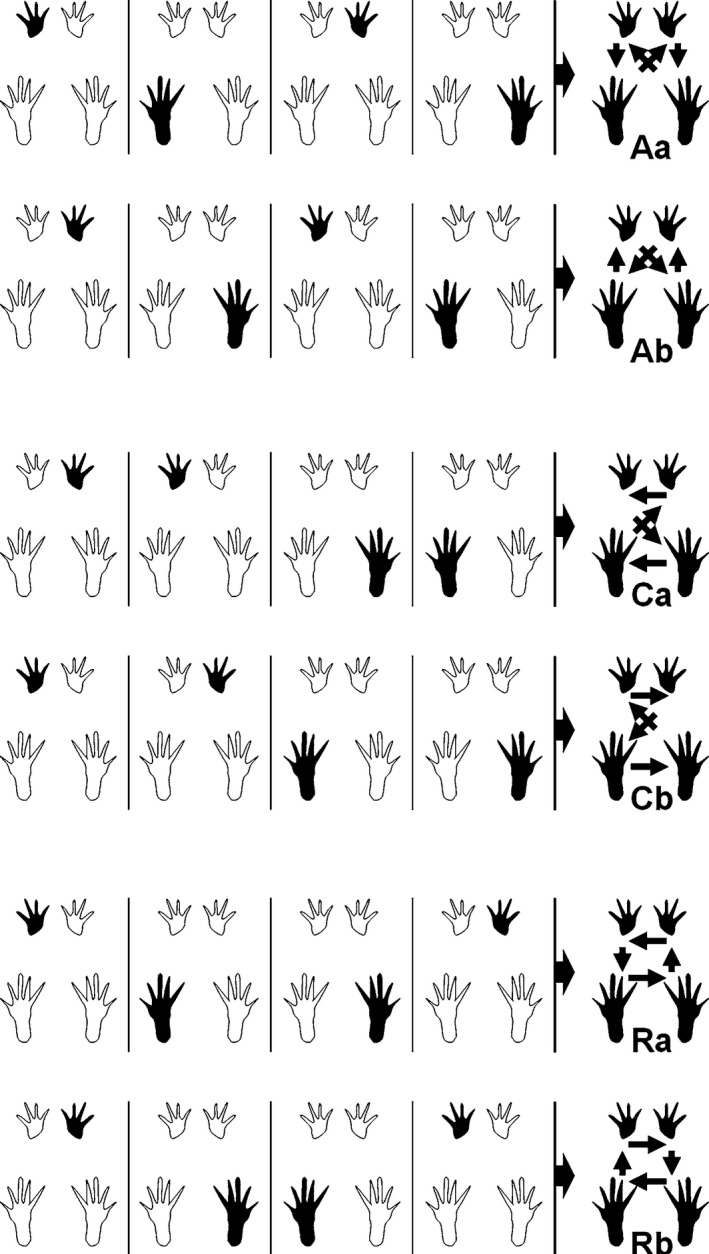
Patterns are either determined by the alternate placement of the right and left paws (Aa and Ab), by the cruciate placement of paws (Ca and Cb), or occur in a rotating pattern (Ra and Rb) (Deumens et al., 2014). Reprinted with permission from Springer Nature

**Figure 4:**
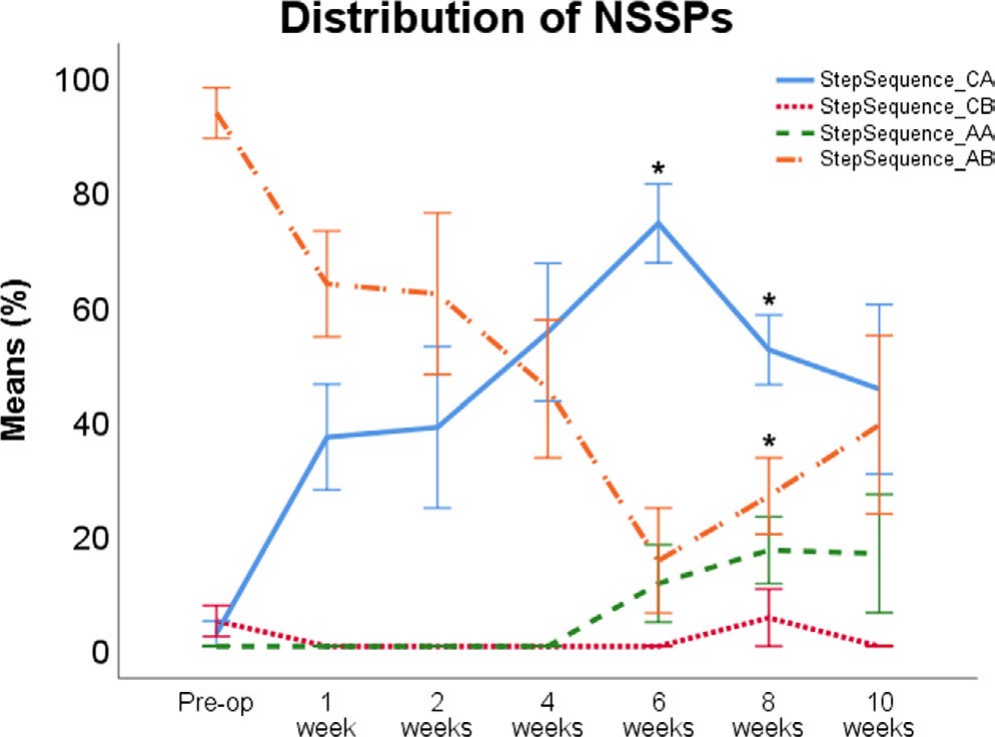
The Ab pattern (orange line) was the predominantly pattern preoperatively. After surgery, usage of this pattern decreased, while the Ca pattern (blue line) was used more often. At WPO6, the Ab pattern reached minimum usage, while the Ca pattern was the most frequently used pattern. Use of the Cb pattern (red line) was reduced to a minimum pre and postoperatively. The Aa pattern (green line) was not used preoperatively, but usage started to increase from WPO4 onward. *p < .05 as compared to preoperative values

#### 3.3.2 Regularity Index

The Regularity Index (not shown) did not reveal any significant changes before and after surgery with a consistent value of ~100%.

#### 3.3.3 Phase dispersions

**Figure 5:**
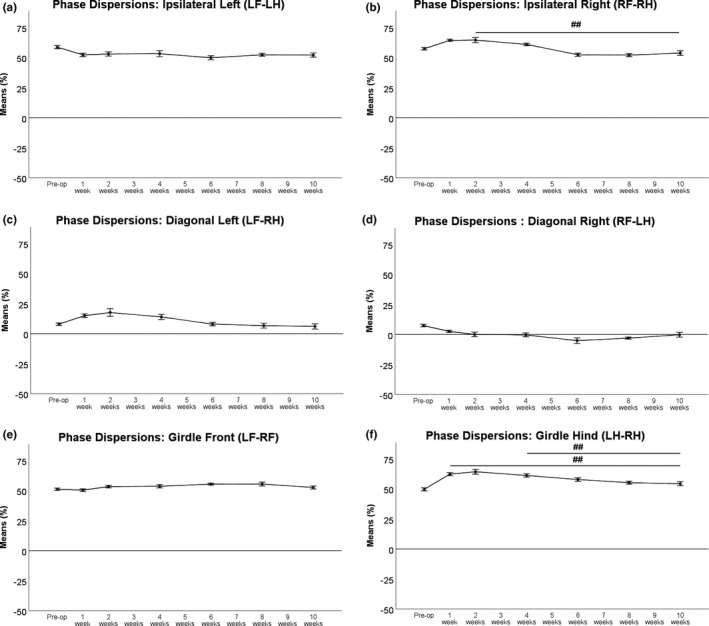
Ipsilateral Left (a) Phase Dispersions were only slightly altered. Ipsilateral Right (b) and Diagonal Left (c) Phase Dispersions were maximally altered at WPO2 while Diagonal Right Phase Dispersions (d) were maximally altered at WPO6, respectively. Ipsilateral Right and Diagonal Left Phase Dispersions returned to baseline at WPO10, but Right Diagonal Phase Dispersions remained slightly decreased in comparison to preoperative values at the end of the observation period. Front Girdle (e) Phase Dispersions also showed only slight changes over time. Hind Girdle Phase Dispersions (f) showed the most accentuated alterations after surgery but returned to baseline at the end of the observation period. *p < .05 as compared to preoperative values, ^##^ *p* < .01

Ipsilateral Left Phase Dispersions (Figure 5a) showed only slight alterations postoperatively. Ipsilateral Right (Figure 5b) and both Diagonal Phase Dispersions (Figure 5c,d) displayed maximal alterations at WPO2 (Figure 5a–f). Ipsilateral Right and Diagonal Left Phase Dispersions reached baseline values at WPO10. Of note, Right Diagonal Phase Dispersions remained slightly decreased in comparison with preoperative values at the end of the observation period. Front Girdle Phase Dispersions (Figure 5e) also showed only minimal changes during the observation period. Hind Girdle Phase Dispersions (Figure 5f) were most strongly altered postoperatively with maximal values reached WPO2. At WPO6, a decrement toward baseline values was observable, and at the end of the observation period, values returned to baseline.

### 3.4 Linear regression analysis of electrophysiology and CatWalk data (Table 2, Figures 6, 7a–h and 6, 7a–d)

**Figure 6:**
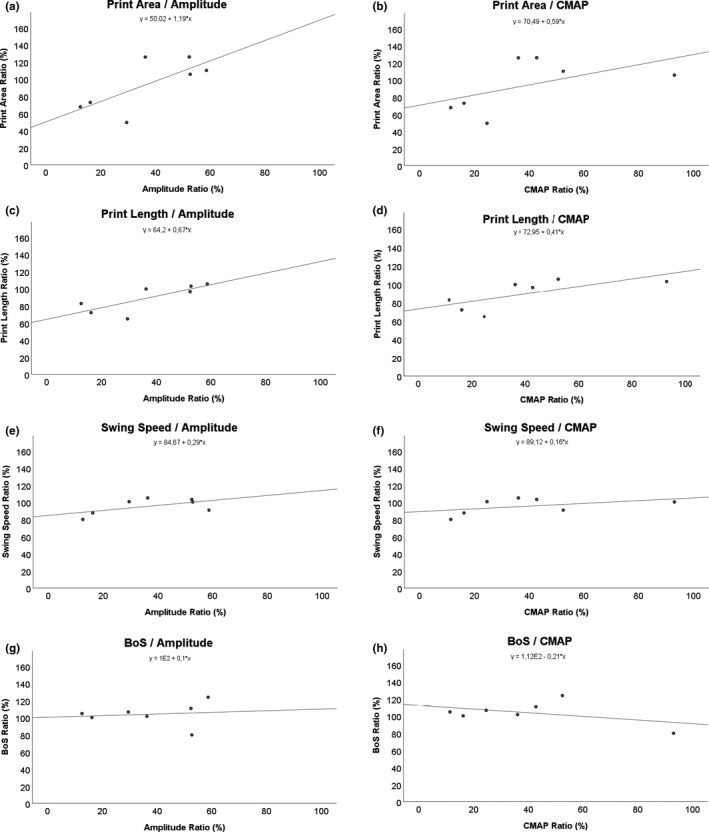
Regression analysis was performed between Amplitude and compound muscle action potential (CMAP) for Print Area (a, b), Print Length (c, d), Swing Speed (e, f), and BoS (g, h). BoS, Base of Support

**Figure 7:**
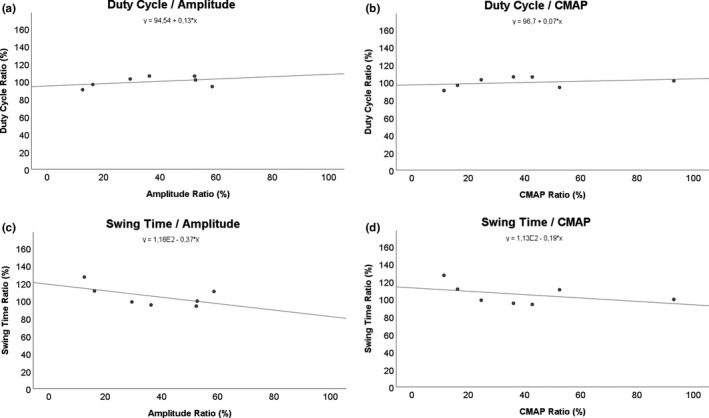
Regression analysis was performed between Amplitude and compound muscle action potential (CMAP) for Duty Cycle (a, b) and Swing Time (c, d)

The *p*‐value and *R* ^2^ of the linear regression analysis for each pair of parameters are shown in Table 2. The linear regression analyses of electrophysiology (amplitude and CMAP) and the general parameters of gait (Figure 6) as well as the pain‐related parameters (Figure 7) revealed that there was only a significant correlation (*p* = .042) between Print Length and the amplitude of the electrophysiological measurements (Figure 6c). Of note, the correlation of Print Area and amplitude (Figure 6a) was close to significant ranges (*p* = .069) as well as the correlation between Print Length and CMAP (Figure 6d; *p* = .078). The three beforementioned pairs of parameters were also the only ones which reached a linear *R* ^2^ close to .5 (Print Length/CMAP) or greater than 0.5 (Print Length − amplitude, Print Area − amplitude).

**Table 2:** *p*‐values and *R* ^2^ of the linear regression analyses

CatWalk data	Amplitude	CMAP
Print Area	*p* = .069 (*R* ^2^ = .517)	*p* = .213 (*R* ^2^ = .290)
Print Length	***p* = .042** (*R* ^2^ = .596)	*p* = .078 (*R* ^2^ = .495)
Swing Speed	*p* = .180 (*R* ^2^ = .327)	*p* = .285 (*R* ^2^ = .222)
BoS	*p* = .776 (*R* ^2^ = .018)	*p* = .317 (*R* ^2^ = .198)
Duty Cycle	*p* = .370 (*R* ^2^ = .162)	*p* = .490 (*R* ^2^ = .100)
Swing Time	*p* = .181 (*R* ^2^ = .325)	*p* = .310 (*R* ^2^ = .203)

Note

Significant *p*‐values (<.05) are marked in bold.

Abbreviations: BoS, Base of Support; CMAP, compound muscle action potential.

## 4 DISCUSSION

The aim of our study was to evaluate whether the CatWalk XT is a feasible tool to evaluate functional recovery in rats with femoral nerve resection and autograft repair. We hypothesized that functional regeneration, assessed via CatWalk XT, would occur faster in the femoral nerve model of the rat than in the sciatic nerve model.

Segmental nerve damage is a very common type of nerve injury but approaches to improve nerve regeneration are difficult to transfer from experimental models in rodents to human patients in general (Tos et al., 2009). Models of segmental nerve injury which lack functional regeneration back to more than 50% of baseline values after nerve resection and repair, like the sciatic nerve model, further impede this transfer of knowledge (Navarro, 2016). The femoral nerve is an alternative and suitable model for peripheral nerve injury. Crucial findings regarding nerve regeneration and neurophysiology were revealed with the help of this model (Brushart, 1988; Brushart, Gerber, Kessens, Chen, & Royall, 1998; Brushart et al., 2002; Irintchev et al., 2018; Kruspe et al., 2014; Madison, Archibald, & Brushart, 1996; Madison, Sofroniew, & Robinson, 2009; Meng et al., 2018; Robinson & Madison, 2004; Tuffaha et al., 2016). Its advantageous features, compared with the sciatic nerve model, include more feasible functional evaluation and absence of limb contractures or automutilation (Irintchev, 2011; Irintchev et al., 2005). Additionally, the femoral nerve caudal to the inguinal ligament only supplies muscular innervation to the quadriceps muscle of the thigh. Since no antagonistic muscle is innervated, misdirection of axons, a severely disadvantageous feature of the sciatic nerve injury model, will have far less impact on functional recovery in the femoral nerve injury model (Haastert‐Talini et al., 2017; Irintchev, 2011).

In 2005, Irintchev et al described single‐frame motion analysis (SFMA), a method to evaluate functional regeneration in mice with femoral nerve injury. This approach was promising, as the group was able to show functional regeneration back to >65% of baseline values after 8 weeks in a 2‐mm resection model of the femoral nerve, bridged with a polyethylene tube (Irintchev et al., 2005). We decided to use the CatWalk XT device instead of the SFMA approach for functional analysis, as we intended to evaluate functional recovery with the same tool used for studies of sciatic nerve injury in rats. On the one hand, SFMA has not been used to study functional regeneration in rats with sciatic nerve injury, and on the other hand, we hoped to provide users of the CatWalk XT, with new insights. Additionally, SFMA requires recording of video data from the side and behind the animal, which is not possible with the CatWalk XT device.

In terms of the general parameters of gait, we observed different individual courses after femoral nerve resection and autograft repair. Print Area decreased moderate in comparison with changes of gait after sciatic nerve resection in comparable studies, which reported a decrement to 15%, but comparable to results after sciatic nerve crush injury (Bozkurt et al., 2011; Deumens et al., 2007). Additionally, restitutio ad integrum was reached 10 weeks after surgery. Print Length and Print Width were not as strongly reduced after surgery and recovered back to >90% at WPO10. Both parameters have been reported to decrease to 40% and 30%, respectively, 1 week after sciatic nerve resection (Deumens et al., 2007). Since studies of segmental sciatic nerve damage involve either no repair (Deumens et al., 2007) or repair of a gap of critical size (Bozkurt et al., 2011), the postoperative course of functional recovery is rather difficult to compare. Full recovery of function was not reported in any study of sciatic nerve resection, with Print Length and Print Width not recovered beyond 50% and Print Area not beyond 20% of baseline values 60 days after surgery (Deumens et al., 2007). However, in models of sciatic nerve crush injury the general parameters of gait recovered back to baseline values 10 weeks after surgery (Bozkurt et al., 2011). We suggest that the decrement of Print Area, Print Length, and Print Width in the femoral nerve model is on the one hand due to decreased overall bodyweight transfer to the respective limb. On the other hand, changed proprioception after saphenous nerve neurotmesis most probably also influences these parameters. In rats as in humans, femoral nerve injury at thigh level leads to impaired knee extension (Pena & Baron, 1988; Stromberg & Hebel, 1976). Impaired knee extension results in plantar hyperflexion in the rat, which in consequence reduces the area of the paw that contacts the floor (Data S2A; Irintchev et al., 2005). In models of segmental sciatic nerve injury, the general parameters of gait are decreased due to impaired hind limb function (Bozkurt, Deumens, et al., 2008; Deumens et al., 2007). Since the sciatic nerve provides muscular fibers to both flexors and extensors of the hind limb, loss of function of these muscle groups seems to be a logical explanation for decreased Print Width, Print Length, and Print Area. Moreover, this can be attributed to misdirected muscular fibers. Another commonly reported problem of the sciatic nerve model, in contrast to the femoral nerve model, is development of neuropathic pain, which further impairs bodyweight transfer to the injured limb (Irintchev, 2011). Additionally, the sciatic nerve supplies sensory fibers to a significant area of the hind limb, in contrast to the femoral nerve, which only releases the saphenous nerve to the hind limb (Stromberg & Hebel, 1976). In conclusion, femoral nerve resection, leading to loss of function of the quadriceps muscle, results in a more moderate impairment of the general parameters of gait than sciatic nerve resection. This is also consistent with the fast recovery of these parameters back to >90% of baseline at WPO10 as reinnervation of the quadriceps muscle results in recovery of knee extension. Stride Length, the product of Swing Time and Swing Speed, was not affected and remained 100% of baseline. Swing Time was significantly increased after femoral nerve resection, whereas Swing Speed was decreased significantly, so their product remained constant. The same phenomenon was observable in rats after sciatic nerve neurotmesis (Bozkurt et al., 2011; Deumens et al., 2007) or induction of sciatic nerve fibrosis (Lemke et al., 2018). An explanation for these findings may be the rats' trait to keep paw placement of both ipsilateral paws on each side almost completely superimposed while proceeding forward (Hruska, Kennedy, & Silbergeld, 1979). By placing each paw in the position of the corresponding ipsilateral paw, rats are able to maintain a save footing and keep their balance during gait. This function is inherent to the central nervous system (Cazalets, Borde, & Clarac, 1995; Zorner et al., 2014). Maintenance of identical Stride Length indicates a supporting mechanism in the case of altered or impaired hind limb function by trying to maintain balance and secure footing during locomotion. Accordingly, Stride Length is altered after central nervous injury and unchanged after peripheral nerve injury in rats (Bozkurt, Deumens, et al., 2008; Hamers et al., 2006, 2001; Lemke et al., 2018). BoS was slightly elevated after surgery and showed only minor variation throughout the entire observation period. This contrasts studies of sciatic nerve resection, where BoS decreased to 50% of baseline after surgery and did not recover back to baseline values (Deumens et al., 2007). This is interesting, since BoS increases after Spinal Cord Injury, to compensate for an instable gait (Hamers et al., 2001; Hendriks et al., 2006; Joosten, Veldhuis, & Hamers, 2004). The decrease in BoS after sciatic nerve resection has been hypothesized to be a separate motor dysfunction rather than a compensatory mechanism by other authors (Deumens et al., 2007). This is a reasonable explanation, but does not exclude a combination of both, which means the rat could still try to counterbalance instable gait by increasing BoS, but this is concealed by the general motor dysfunction of the hind limb. This general motor dysfunction of the hind limb after femoral nerve injury may not be as severe as after sciatic nerve injury, and therefore, BoS was only slightly altered after femoral nerve resection and autograft repair. This also fits with our theory that the onset of neuropathic pain in sciatic nerve injury models affects most of the parameters of gait. One could conclude that rats try to keep an injured limb close to their body's center while walking, especially if they experience pain or discomfort in it. A reasonable explanation for the overall more stable course of BoS in our model could be that the onset of neuropathic pain is at least very uncommon or even not existing in the femoral nerve model, as mentioned before.

The pain‐related parameters of gait, Duty Cycle, Swing Time, and Stand Time changed significantly after femoral nerve resection and autograft repair, were maximally altered from baseline values at WPO2 and returned to 80%–90% of baseline around 6–8 weeks after injury. Ten weeks after surgery, over 90% of the baseline values were reached. These findings are in accordance with studies on sciatic nerve crush injury (Bozkurt, Deumens, et al., 2008; Bozkurt et al., 2011) and sciatic nerve resection (Deumens et al., 2007) in terms of the initial change in these parameters after nerve surgery. Of note, femoral nerve resection seems to produce more moderate changes of the pain‐related parameters. These parameters do not recover to preoperative values after sciatic nerve resection and autograft repair in rats, but to 80% around 6 weeks after crush injury (Bozkurt et al., 2011). This suggests that the impact of pain on these parameters must be comparable between femoral nerve transection and crush injury. However, it also contradicts the assumption that neuropathic pain does not develop in the femoral nerve injury model (Irintchev, 2011). Duty Cycle, Swing Time, and Stand Time were correlated with mechanical allodynia by Vrinten and Hamers in 2003, after inducing a chronic constriction injury of the sciatic nerve (Vrinten & Hamers, 2003). All three parameters correlated with von Frey testing for mechanical allodynia, supported by other studies on locomotor behavior in rats with mechanical allodynia (Kim & Chung, 1992; Min et al., 2001; Seltzer, Dubner, & Shir, 1990). However, Deumens and colleagues concluded that decreased weight loading due to pain at least contributes to the observed changes (Medhurst et al., 2002; Schott et al., 1994). Agreeing with them, we hypothesize that changes to the pain‐related parameters of gait are not solely related to pain in the femoral nerve model, but to decreased body weight transfer, with the underlying mechanisms discussed earlier. This is also affirmed by the, although moderate, decrease in Duty Cycle after isovolumetric muscle loss of the quadriceps muscle. It is safe to exclude neuropathic pain in this setting (Li et al., 2014). Lastly, mechanical allodynia has been reported to develop with a delay of 14 days after sciatic nerve injury, contrasting the immediate impairment of locomotor behavior (Deumens et al., 2007). Interestingly, in our study the pain‐related parameters were also maximally affected by femoral nerve neurotmesis at WPO2. This, however, may not be related to the onset of mechanical allodynia, but due to collateral sprouting of sensory fibers of the sciatic nerve, starting to innervate the area of the transected saphenous nerve. Collateral sprouting of the saphenous nerve after transection of the sciatic nerve has been reported previously (Kambiz et al., 2015). Of note, this collateral sprouting seems to be limited to high‐threshold nociceptive fibers (Barbay, Peden, Falchook, & Nudo, 2002). If the same is true for collateral sprouting of the sciatic nerve after femoral nerve injury, it would explain the noted effect on the pain‐related parameters of gait 2 weeks after surgery.

The coordination‐related parameters illuminate functional deficits after nerve injury and how they are compensated for from another perspective. The Regularity Index remained unchanged at 100% after femoral nerve resection and autograft repair. This is in accordance with findings after sciatic nerve transection. It is safe to assume that femoral nerve resection does not impair hind limb‐forelimb coordination itself, as observable after spinal cord injuries, since the general pattern generator is located within the central nervous system (Alford & Schwartz, 2003; Bhimani et al., 2017; Hamers et al., 2006; Kyriakou et al., 2016). The distribution of NSSP was significantly altered after femoral nerve resection, with a significant decrease in Ab patterns. This was compensated for by an increasing amount of the Cruciate Pattern Ca and, to a lesser extent, increased use of the Aa pattern starting from WPO4 onward. Use of the cruciate pattern has been reported to be a compensatory mechanism to reduce burden on the affected hind limb (Deumens et al., 2014). This is well explained by analyzing the footfall pattern of the Cruciate b (Cb) pattern: LF‐RF‐LH‐RH. Use of this NSSP leaves the injured limb to be the last to be placed during the walking cycle and enables the rat to explore the ground with both front paws first, before placing the hind paws. In both the Ab and Ca patterns, the right hind limb is preceded by the most distant front paw (e.g., left front paw), as a compensatory mechanism to maximize the distance between the body's center of gravity and the affected hind paw (Deumens et al., 2007). We hypothesize, that the Ab pattern initially remains the most used pattern postoperatively, because the rats were used to use this pattern before surgery. These altered paw placements started to normalize later after femoral nerve injury than changes of individual paw function. At the end of our 10‐week observation period, there was still no return of the NSSP distribution back to preoperative values. A possible explanation for this phenomenon could be altered proprioception of the right hind limb resulting in a different step sequence pattern in compensation. This relates more likely to initial hypesthesia due to resection of the saphenous nerve given the missing reports of neuropathic pain after femoral injury in rats (Irintchev, 2011). We also consider collateral sprouting of sensory fibers of the sciatic nerve as contributing factor. Since collateral sprouting of sensory fibers alters the sensory areas of the respective nerves (e.g., sural and saphenous nerve), intracerebral representation of these areas is also altered (Barbay et al., 2002). This could result in altered proprioception of the respective limb, explaining the observed findings. The increase in these compensatory NSSP plateaued around WPO6–WPO8 and started to decrease afterward but did not return to baseline values at the end of the observation period. We therefore conclude that return of proprioception and onset of “normal” sensation of the hind limb returns later after femoral nerve resection and repair than motor function. This hypothesized delay is mirrored by the course of general and pain‐related parameters of gait, which returned to preoperative values around WPO10. In our opinion, further studies should be conducted to investigate the long‐term course of NSSP after femoral nerve injury in rats, since no data have been published so far. This could grant new insights about central compensatory mechanism for peripheral nerve injury in rodents. To our judgment, alterations of the Phase Dispersions after femoral nerve injury are also compensatory mechanisms for impaired hind limb function. As mentioned before, rats try to maximize the distance between their center of gravity and the affected hind limb. In our study, Hind Girdle, Right Ipsilateral, and Left Diagonal Phase Dispersions increased after femoral nerve resection and autograft repair. This is consistent with findings after sciatic nerve crush injury. In this injury model, Couplings were used instead of Phase Dispersions, but consistent with our observations, changes were most accentuated immediately after surgery and the values returned to baseline around 5–6 weeks after surgery (Bozkurt, Deumens, et al., 2008). Alterations of the Hind Girdle and Diagonal Phase Dispersions were reported after sciatic nerve resection, but these changes were much more accentuated (Deumens et al., 2007). Since placement of the other three paws was not altered temporally, the Front Girdle, Left Ipsilateral, and Right Diagonal showed little variation over the time course of 10 weeks in our study. In conclusion, temporal alterations of paw placement after femoral nerve resection and autograft repair, as displayed by the Phase Dispersions, are consistent with findings reported after sciatic nerve injury before. In our opinion, they should be thought of as counterbalance and regulatory mechanisms in combination with changes in NSSP distribution, to decrease burden on the hind limb after nerve injury. Since changes are less accentuated after femoral nerve injury, we hypothesize this injury model to have less impact on motor performance in rats than models of sciatic nerve resection injury.

Linear regression analysis to evaluate correlations between the CatWalk data and electrophysiologic measurements revealed that only Print Length and the amplitude of the electrophysiological measurements showed statistically significant correlation. Other authors have published their findings on such correlation analysis between functional data and electrophysiology in the past, indicating that both do not necessarily correlate (Kappos et al., 2017). However, Print Area and amplitude as well as Print Length and CMAP revealed correlation close to statistically significant levels. This is in accordance with the beforementioned considerations of how loss of function of the quadriceps muscle reduces the print area and print length of the paw, respectively. However, it is of interest, that all other parameters showed no statistically significant correlation in our study. On the other hand, these findings indicate that the femoral nerve, by means of the quadriceps muscle of the thigh, is only marginally contributing to the swing speed and duration of the swing phase of the hind paw in rats, strongly contrasting the sciatic nerve and the plethora of muscles innervated by it.

The sciatic nerve model, although widely used, has several major limitations which hamper its utility and significance of results. Among these are the high incidence of limb contractures and automutilation as well as the onset of neuropathic pain (Irintchev, 2011; Tos et al., 2009). Additionally, the severity of the nerve injury plays a major role in assessment of functional recovery after sciatic nerve injury, since full recovery to baseline seems only to occur in models of crush injury (axonotmesis) and, as recently proven, in models of epineural fibrosis (Bozkurt, Deumens, et al., 2008; Bozkurt et al., 2011; Lemke et al., 2018). In contrast, most of the parameters of gait merely reached around 50% of baseline 10 weeks after sciatic nerve resection and autograft repair (Bozkurt et al., 2011; Deumens et al., 2007). Any injury more severe than crush injury seems to limit regenerative capability to a maximum of 40%–60% in the rat sciatic nerve model (Hare et al., 1992). In conclusion, potential benefits or other effects of an experimental approach may be superimposed by the natural limitation of functional recovery in such a model. This is of serious concern regarding the necessary observation to investigate functional outcome in this setting. This is especially relevant in the context of neuropathic pain experienced by the animals (Tos et al., 2009).

Comparison of our work with publications on gait analysis in rats with segmental sciatic nerve injury is difficult, as there are major differences regarding distance from lesion to target organ, method of repair, and, most importantly, length of the nerve gap (Kappos et al., 2017). However, besides these considerations, we were able to report almost full functional regeneration at 10 weeks after segmental nerve damage, in contrast to the sciatic nerve defect model as published by various authors (Hare et al., 1992; Irintchev, 2011; Lee et al., 2013; Perussi Biscola, Politti Cartarozzi, Ferreira Junior, Barraviera, & Leite Rodrigues de Oliveira, 2016).

On the other hand, very fast nerve regeneration may also be disadvantageous, as effects of any specific treatment or therapeutic approach may unfold at a time point when full regeneration has already occurred. However, in our model of a 6‐mm femoral nerve gap, reconstructed with an autologous graft, functional recovery to preoperative levels occurred 10 weeks after surgery. We believe this to be a reasonable evaluation period. Of note, the femoral nerve model also bears several limitations. Observations regarding neural regeneration in rodents are very difficult to adapt to human patients (Gordon, 2016; Kaplan, Mishra, & Kohn, 2015; Seddon, Medawar, & Smith, 1943; Sjoberg & Kanje, 1990). In this specific setting, it is even more difficult, as the fast rate of functional regeneration limits long‐term observation. The amount of data obtainable when investigating the effects of a specific treatment or therapeutic approach is also restricted. On the other hand, the regenerative potential and easy assessment of functional recovery is disclosed as one of the big advantages of the femoral nerve model.

Finally, we want to stress the importance of objective and precise data acquisition. One limitation of our study was the limited statistical power due to insufficiently adjusted camera settings. On top of that some of the video, data were blurred due to a moist walkway since the rats had contaminated the CatWalk with their urine (Data S2B). We strongly like to point out these problems since they can hamper the validity of any study using the CatWalk device.

In this study, we identified several parameters of gait which allow precise measurements of functional recovery in a rodent model of peripheral nerve resection and autograft repair. Therefore, use of the CatWalk XT for complex evaluation of gait in the rat model of femoral nerve resection and autograft repair provides researchers with a feasible tool to monitor the full extent of their new therapeutic approach or treatment within a tenable postoperative observation period. As changes of gait after femoral nerve injury were unstudied with the CatWalk XT before, our study grants important new insights for researchers interested in in vivo models of peripheral nerve injury and regeneration.

## CONFLICT OF INTEREST

None declared.

## AUTHORS' CONTRIBUTIONS

J. C. H. and D. H. performed the gait analysis, analyzed the data, and wrote the manuscript. H. R. conceptualized the work and critically revised the manuscript. All authors read and approved the final version of the manuscript.

## Data Availability

The data that support the findings of this study are available from the corresponding author upon reasonable request.
