# Supplementary material for: The course of recovery of locomotor function over a 10‐week observation period in a rat model of femoral nerve resection and autograft repair
Source: Brain Behav. 2020 Feb 25;10(4):e01580. doi: 10.1002/brb3.1580 (PMC7177579; doi:10.1002/brb3.1580)
Supplement: Supplementary file 1 [file BRB3-10-e01580-s001.pdf]

Parameter of gait	**Observed Power** (*n = 5)*
Print Area	>0.8
Print Length	>0.8
**Stride Length**	**<0.8 (0.042)**
Base of Support	>0.8
Duty Cycle	>0.8
Swing Time	>0.8
Swing Speed	>0.8
**Regularity Index**	**<0.8 (0.799)**
Phase Dispersions Left Ipsilateral	>0.8
Phase Dispersions Right Ipsilateral	>0.8
Phase Dispersions Left Diagonal	>0.8
Phase Dispersions Right Diagonal	>0.8
Phase Dispersions Front Girdle	>0.8
Phase Dispersions Hind Girdle	>0.8
Step Sequence Aa	>0.8
Step Sequence Ab	>0.8
Step Sequence Ca	>0.8
Step Sequence Cb	>0.8
Step Sequence Ra	not calculated*
Step Sequence Rb	not calculated*

Supplementary data 1 - Observed power as calculated post-hoc for ANOVA with repeated measures with SPPS
*observed power was not calculated as there was no difference to the baseline value at any time point.
