# Supplementary material for: The course of recovery of locomotor function over a 10‐week observation period in a rat model of femoral nerve resection and autograft repair
Source: Brain Behav. 2020 Feb 25;10(4):e01580. doi: 10.1002/brb3.1580 (PMC7177579; doi:10.1002/brb3.1580)
Supplement: Supplementary file 2 [file BRB3-10-e01580-s002.pdf]

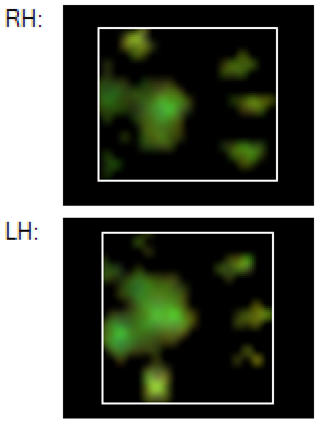

Figure 2A-1 *– Print Area of the right (top) and left hind paw (bottom) preoperatively*

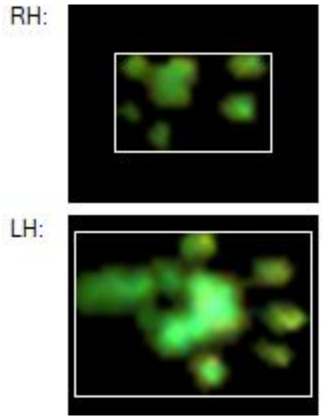

Figure 2A-2 *– Print Area of the right (top) and left hind paw (bottom) at WPO1*

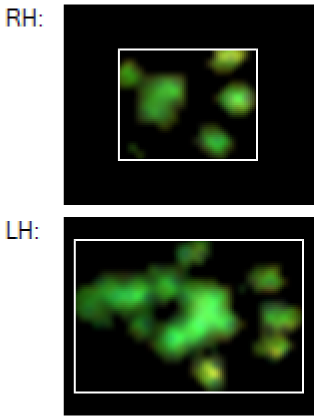

Figure 2A-3 *– Print Area of the right (top) and left hind paw (bottom) at WPO2*

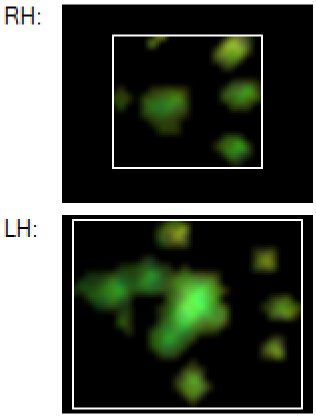

Figure 2A-4 *– Print Area of the right (top) and left hind paw (bottom) at WPO4*

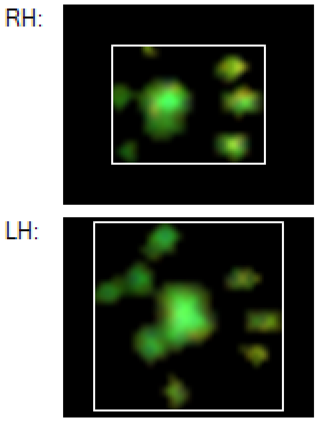

Figure 2A-5 *– Print Area of the right (top) and left hind paw (bottom) at WPO6*

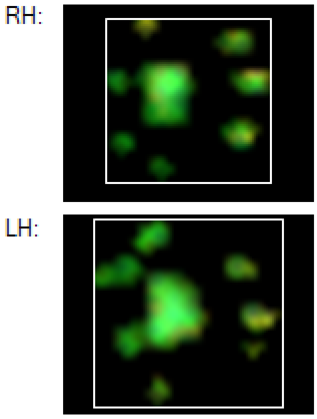

Figure 2A-6 *– Print Area of the right (top) and left hind paw (bottom) at WPO8*

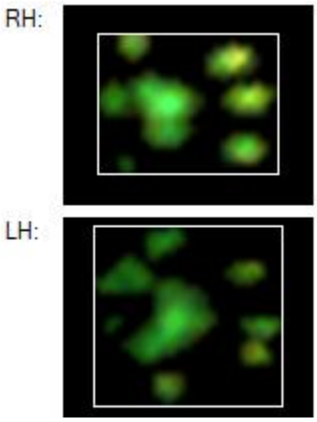

Figure 2A-7 *– Print Area of the right (top) and left hind paw (bottom) at WPO10*
