# Supplementary material for: The course of recovery of locomotor function over a 10‐week observation period in a rat model of femoral nerve resection and autograft repair
Source: Brain Behav. 2020 Feb 25;10(4):e01580. doi: 10.1002/brb3.1580 (PMC7177579; doi:10.1002/brb3.1580)
Supplement: Supplementary file 3 [file BRB3-10-e01580-s003.pdf]

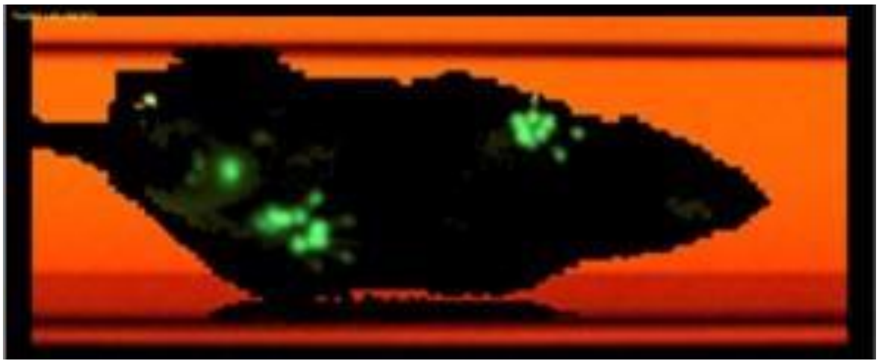

Figure 2B-1 *– CatWalk data of an animal which contaminates the walkway with urine during walking*

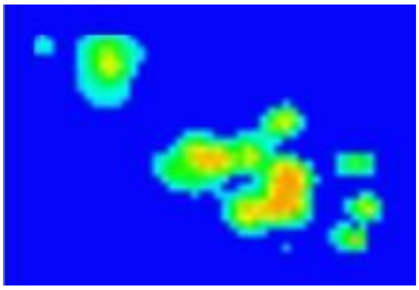

Figure 2B-2 *– Pawprint Picture of Figure 2B-1*

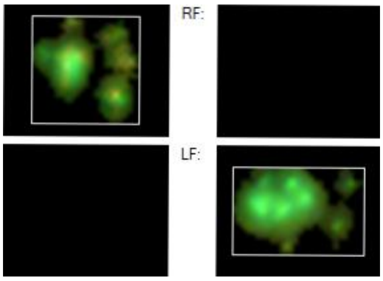

Figure 2B-3 *– Blurred paw prints*

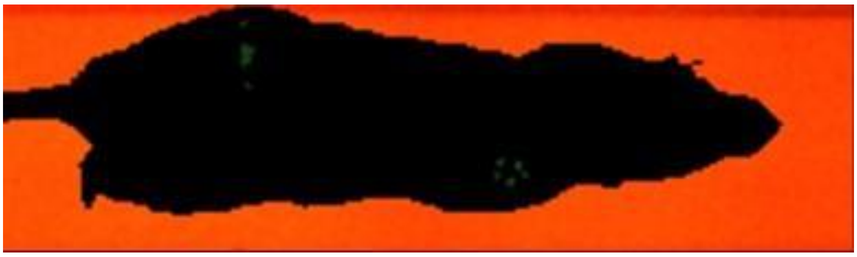

Figure 2B-4 *– CatWalk data of an animal which paws prints are only faintly visible due to insufficient camera settings*

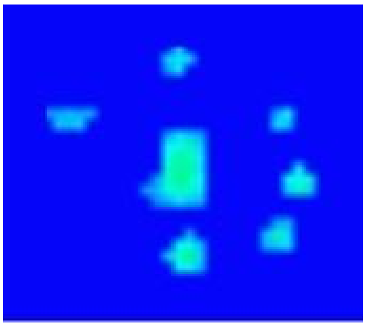

Figure 2B-5 *– Pawprint Picture of Figure 2B-4*
